# The synthetic glycolipid-based TLR4 antagonist FP7 negatively regulates *in vitro* and *in vivo* haematopoietic and non-haematopoietic vascular TLR4 signalling

**DOI:** 10.1177/1753425918798904

**Published:** 2018-09-12

**Authors:** Charys Palmer, Francesco Peri, Frank Neumann, Feroz Ahmad, David S. Leake, Grisha Pirianov

**Affiliations:** 1 Department of Biomedical and Forensic Sciences, Anglia Ruskin University, Cambridge, UK; 2 Department of Biotechnology and Biosciences, University of Milano-Bicocca, Italy; 3 Innaxon, Tewkesbury, UK; 4 School of Biological Sciences and Institute of Cardiovascular and Metabolic Research, University of Reading, Reading, UK

**Keywords:** TLR4, TLR4 antagonist FP7, vascular inflammation, oxidised low-density lipoproteins, haematopoietic cells and non-haematopoietic cells

## Abstract

TLRs, including TLR4, have been shown to play a crucial role in cardiovascular inflammatory-based diseases. The main goal of this study was to determine the potential of FP7, a synthetic glycolipid active as a TLR4 antagonist, to modulate haematopoietic and non-haematopoietic vascular TLR4 pro-inflammatory signalling. HUVEC, human THP-1 monocytes, THP-1-derived macrophages, mouse RAW-264.7 macrophages and Angiotensin II-infused apolipoprotein E-deficient mice were *in vitro* and *in vivo* models, respectively. Western blotting, Ab array and ELISA approaches were used to explore the effect of FP7 on TLR4 functional activity in response to bacterial LPS (*in vitro*) and endogenous ligands of sterile inflammation (*in vitro* and *in vivo*). Following activation of TLR4, *in vitro* and *in vivo* data revealed that FP7 inhibited p38 MAPK and p65 NF-kB phosphorylation associated with down-regulation of a number of TLR4-dependent pro-inflammatory proteins. In addition to inhibition of LPS-induced TLR4 signalling, FP7 negatively regulated TLR4 activation in response to ligands of sterile inflammation (hydroperoxide-rich oxidised LDL, *in vitro* and Angiotensin II infusion, *in vivo*). These results demonstrate the ability of FP7 to negatively regulate *in vitro* and *in vivo* haematopoietic and non-haematopoietic vascular TLR4 signalling both in humans and mice, suggesting the potential therapeutic use of this TLR4 antagonist for pharmacological intervention of vascular inflammatory diseases.

## Introduction

The worldwide incidence of cardiovascular diseases (CVD) has increased dramatically for the last few decades because of a variety of health, economic and social factors.^1^ At the same time there is a lack of direct pharmacological prevention or treatment of CVD. Discovery of drugs for treatment and stabilisation of these diseases is a worthy challenge, with a significant commercial impact to relieve a significant global financial burden from the health services. The pathogenesis of CVD is complex, involving the interaction of several fundamental physiological processes; however, it is believed that sterile inflammation plays a fundamental role in all stages of CVD.^2^ TLRs serve as PRRs within the immune system and recognise PAMPs and danger-associated molecular patterns (DAMPs) ligands as inflammatory triggers. Among these receptors, TLR4 is known to be activated by the Gram-negative bacteria LPS. Additionally, TLR4 is also activated by endogenous DAMPs, known as ligands of sterile inflammation, such as heat-shock proteins,^3^ fibronectins, small fragments of hyaluronan,^4^ saturated fatty acids^5^ and oxidised low-density lipoprotein (oxLDL).^6^ TLR4 expression has been described both in haematopoietic and non-haematopoietic cells.^6^ Moreover, TLR4 has been documented to be implicated in the pathogenesis of inflammatory-related CVD. For example, recent studies have demonstrated that deletion of the *TLR4* gene in haematopoietic and non-haematopoietic cells protected against CVD.^7–10^ These findings strongly support the idea that regulation of TLR4 may be a novel target for therapeutic control of CVD.

Over the last two decades TLR4 antagonists have been evaluated in preclinical and clinical studies; however, none have been approved for clinical use for the time being.^11,12^ Therefore, discovery of novel TLR4 modulators is a big challenge with high commercial and social impact. Recently, we have developed a synthetic anionic glycolipid, named FP7, as a TLR4 antagonist.^13^ FP7 is an MD-2 ligand that binds the hydrophobic cavity of MD-2 and displaces LPS and other ligands, thus inhibiting TLR4 activation (formation of TLR4/MD-2/LPS homodimer).^14,15^

The main aim of this study was to investigate the potential of FP7 to modulate human and mouse vascular TLR4 signalling. Our results determined the ability of FP7 to negatively regulate non-haematopoietic and haematopoietic TLR4 signalling, suggesting the potential therapeutic use of this TLR4 antagonist for treatment of inflammatory CVD.

## Materials and methods

### Materials

FP7 was prepared in F. Peri laboratories (University of Milano Bicocca) by multistep organic synthesis, and the purity and identity of the compound was assessed by NMR, mass spectrometry and HPLC analyses as previously described.^15^ LPS [*Salmonella* Minnesota (Re) R595, TLRpure™] was kindly provided by Innaxon, Tewkesbury. For *in vitro* experiments FP7 was reconstituted in DMSO/ethanol (1:1). For *in vivo* experiments FP7 was reconstituted in Lipodisq™, a biodegradable liposomal nano-disc formulation (Malvern Cosmeceutics, Malvern, UK). FP7 in Lipodisq™ was formulated and prepared by Innaxon, Tewkesbury.

### Preparation of lipid hydroperoxide-rich LDL

Native LDL (2 mg protein/ml) was dialysed at 4°C against phosphate buffer (140 mM NaCl, 8.1 mM Na_2_HPO_4_, 1.9 mM NaH_2_PO_4_, and pH 7.4), to remove residual EDTA, followed by dialysis for 24 h against MOPS buffer (10 mM MOPS, 150 mM NaCl, and pH 7.4, treated with washed Chelex-100). The LDL was then oxidised by dialysis against MOPS buffer containing 10 μM CuSO_4_ (both within the bag and in the surrounding dialysis buffer) for 24 h at 4°C to form hydroperoxide-rich LDL. Oxidation was stopped by the addition to the dialysis bag of 1 mM EDTA and the LDL dialysed for a further 24 h against phosphate buffer containing 100 μM EDTA, filter-sterilised and stored at 4°C.^16^

### Cells maintenance and treatment

HUVEC, purchased from Promocell (Heidelberg, Germany), were treated in accordance to the company’s instructions. The cells were maintained at 37°C, 5% constant atmospheric condition of CO_2_ in endothelial cell growth medium 2 (Promocell) in 25 cm^2^ flasks pre-coated with 1% attachment factor (Sigma, UK). HUVEC were used between passages 3 and 5. THP-1 cells were obtained from the European Collection of Animal Cell Cultures (Salisbury, Wiltshire, UK) and cultured in Roswell Park Memorial Institute (RPMI) (+10% heat-inactivated FBS (HIFBS), +1% glutamine, +1% penicillin/streptomycin). Cells were split three times weekly and maintained at a density of ∼0.3 × 10^6^ cells/ml. For differentiation of THP-1 cells 25 nM of phorbol 12-myristate 13-acetate (PMA) was added to plated cells for 3 d before washing three times with fresh medium. Cells were then left to rest overnight (16 h) before treatment. Mouse RAW-264.7 macrophages were obtained from Prof Z. Mallat (Cambridge University, Cambridge, UK) and cultured in DMEM (+10% HIFBS, +1% penicillin/streptomycin). Medium was changed three times weekly and cells split after reaching 60% to 70% confluence.

All cells were pre-treated with FP7 (0–10 μM) for 1 h, then exposed to LPS (10 or 100 ng/ml) or hydroperoxide-rich LDL (0–100 μg/ml protein) for 1 or 16 h.

### Animal model

All animal experiments were approved by the local animal research work ethical review board at St George’s, University of London. Twelve 3-mo-old Apolipoprotein *(Apo) E^–/–^/C57Bl6* were randomly separated into three groups.^17^ Two groups (*n* = 4) were infused with Angiotensin II (1 μg/min/kg), and the third (*n* = 4) was infused with saline. FP7 was co-administered s.c. (3 mg/kg/d, 50 μl Lipodisq™) and the remaining two groups were co-administered s.c. with drug vehicle (50 μl Lipodisq™). At d 3, following Angiotensin II infusion, animals were sacrificed.

### Tissue collection and processing

Termination of the experiments was scheduled at d 3 after insertion of the osmotic pumps. Animals were transcardially perfused, at physiological pressures, with PBS containing a cocktail of proteinase inhibitors (Sigma Aldrich, UK) for 10 min at 4°C, after which the aortic tree was dissected at 4°C, removing the loose connective tissue around the exterior of the artery. The suprarenal segment of the mouse aorta was harvested, frozen in liquid nitrogen, and stored at –80°C until processed. Tissues were ground to a fine powder under liquid nitrogen and then lysed by sonication in a non-denaturing phosphate lysis buffer [20 mM sodium phosphate, 137 mM NaCl, 25 mM sodium β-glycerophosphate, 2 mM sodium pyrophosphate, 2 mM EDTA, 10% glycerol, 1% Triton X-100 and protease inhibitor cocktail (Sigma-Aldrich, UK)]. Cell lysates were incubated on ice for 20 min and centrifuged for 20 min at 10,000 *g* at 4°C. Supernatants were removed and the pellet of insoluble material discarded. Protein concentration of the cell supernatant was determined by the bicinchoninic acid method (Pierce, Rockford, IL, USA).

### Western blot analysis of protein expression and phosphorylation

Cell or tissue lysates (10–20 μg) were separated on a 7.5% TGX gel and transferred to polyvinyldifluoride membranes (Bio-Rad, UK) and blocked using 5% (wt/vol) skimmed milk in TBS/0.1% (v/v) Tween-20 for 1 h at room temperature (20°C). Blots were incubated overnight at 4°C with primary Abs: phospho-p38 (4511), phospho-p65 NF-kB (3031), phospho-JNK (9255) and β-actin (12262) from Cell Signalling Technology (NEB, Herts, UK) (1:1000 dilution in TBS, 1% milk). After washing in TBS/0.1% (v/v) Tween-20, blots were incubated with HRP-conjugated Ab at room temperature for 1 h in TBS/0.1% (v/v) Tween-20 and 5% milk. After the final wash, immunoreactivity was visualized using the chemiluminescent substrate ECL Plus (Healthcare, Bucks, UK). Densitometric analysis was performed using G-box software Genetools 4.3.8. (Synoptics UK). The level of cellular actin was used as a loading control.

### ELISA

Human monocyte chemoattractant protein (MCP)-1, IL-1β, IL-6, IL-8 and mouse macrophage inflammatory protein (MIP)-1γ production were measured in cell and tissue lysates (20 μg protein) or cell culture medium (10–250 μl) using ELISA kits (RayBiotech, USA) following the manufacturer’s instructions. At the final stage absorbance was measured at 450 nm using a microplate reader Tecan v7.1 (Sunrise, Austria). Protein concentration was determined using the GraphPad Prism version 7.01.

### Statistical analysis

Data were reported as mean ± SD and analysed with one-way ANOVA followed by the post-hoc Tukey test for multiple comparisons using GraphPad Prism version 7.01. A value of *P* < 0.05 was considered significant.

## Results

### FP7 inhibits LPS-stimulated TLR4 signalling in human THP-1 cells

**Figure 1.**
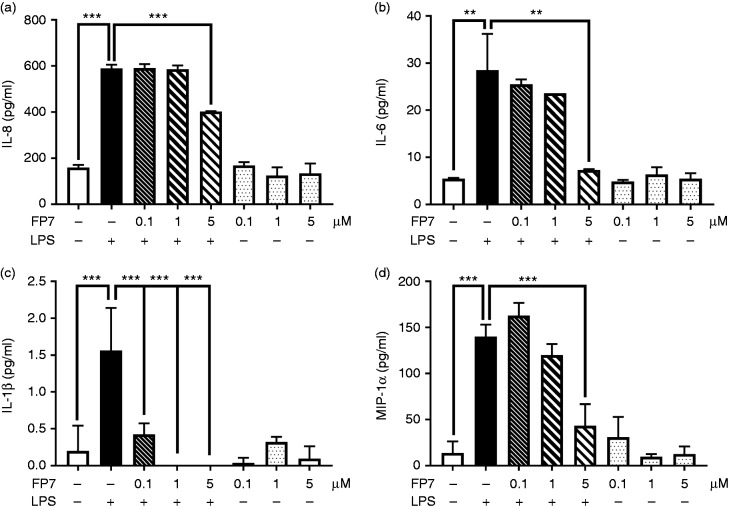
FP7 down-regulates LPS/TLR4-induced cytokine production in THP-1 monocytes. THP-1 monocytes were pre-incubated with FP7 (0–10 μM) for 1 h and then exposed to LPS (100 ng/ml) for 16 h. ELISA was used to measure cytokine production: IL-8 (a), IL-6 (b), IL-1β (c) and macrophage inflammatory protein (MIP)-1α (d) following 16 h exposure to LPS. Data are mean ± SD, *n* = 3 at each data point. Significant results are indicated as *P < 0.05, **P < 0.01 and ***P < 0.001.

TLR4 signalling has been shown to play a critical role in the functional activity of immune-competent cells at any stage of the inflammatory process. It is clear that prolonged activation of the receptor as a result of the bioavailability of PAMPs and DAMPs can lead to chronic inflammation that is associated with development and progression of inflammatory diseases. To study the effect of FP7 on TLR4 signalling in monocytes, we utilised THP-1 cells as an *in vitro* cell model. We investigated the potential of FP7 to modulate the expression of LPS/TLR4-dependent proteins. ELISA results demonstrated that FP7 negatively regulated the production of a number of LPS/TLR4-driven pro-inflammatory proteins (IL-8, IL-6, MIP-1α and IL-1β) in a concentration-dependent manner in THP-1 cells (Figure 1a–d). These data showed the ability of FP7 to effectively reduce TLR4 signalling in THP-1 human monocytes.

### FP7 suppresses both LPS and hydroperoxide-rich LDL-induced TLR4 signalling in human THP-1-derived macrophages

**Figure 2.**
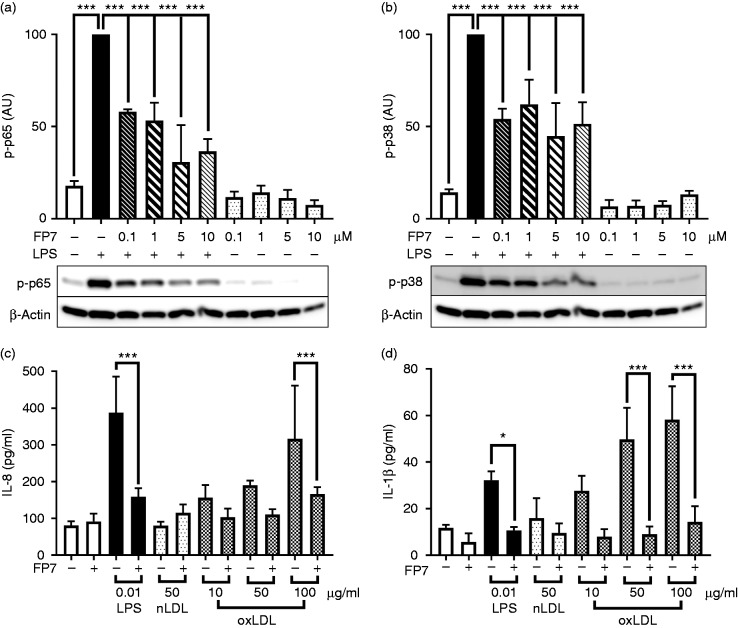
FP7 inhibits LPS and hydroperoxide-rich oxidised LDL (oxLDL)-induced TLR4 signalling in THP-1-derived macrophages. Cells were differentiated with PMA and pre-incubated with FP7 (0–10 μM) for 1 h and then exposed to LPS (10 ng/ml) for 60 min. Western blot was used to measure p65 NF-kB (a) and p38 MAPK (b) phosphorylation. Actin was used as a loading control. THP-1 macrophages were exposed to LPS (10 ng/ml), native low-density lipoprotein (LDL) (nLDL, 100 µg/ml protein) or oxLDL( 0–100 µg/ml protein) in the presence or absence of FP7 (10 µM). IL-8 (c) and IL-1β (d) production was measured in the medium after 16 h via ELISA. Data are mean ± SD, *n* = 3 at each data point. Significant results are shown as *P < 0.05 and ***P < 0.001.

Macrophages are important immune cells in maintaining tissue integrity and culminating the immune responses in health and diseases. Plasticity and flexibility are key features of macrophages and their activation status was shown to be regulated by TLR4 signalling.^18^ To determine the effect of FP7 on LPS-induced TLR4 signalling in THP-1-derived macrophages, we analysed the activation of p38 MAPK/p65 NF-kB as second messengers in TLR4 signalling and production of several TLR4-dependent pro-inflammatory proteins released from activated THP-1-derived macrophages. Initially, we tested the ability of FP7 to modulate TLR4 signalling in THP-1-derived macrophages in response to LPS. Immunoblotting results showed that pre-treatment of THP-1 cells with FP7 (0–10 μM) significantly inhibited LPS-induced p38 MAPK and p65 NF-kB phosphorylation in a concentration-dependent manner (Figure 2a and b). Next, we measured the production of TLR4-dependent proteins in the presence or absence of FP7. As illustrated in Figure 2 (c and d), ELISA results demonstrated that FP7 negatively regulated the production of LPS/TLR4-driven IL-8 and IL-1β pro-inflammatory proteins in THP-1-derived macrophages. To further investigate the effect of FP7 on cytokine production, this compound was administered prior to, simultaneously with and after LPS in THP-1-derived macrophages. Irrespective of the time of administration, FP7 greatly inhibited LPS-induced IL-6 and TNF-α production in THP-1 macrophages (Supplemental Figure 2). These data showed the ability of this small molecule to reverse LPS/TLR4/cytokine production *post factum*.

It is well documented that chronic sterile inflammation is the major contributor to development of CVD.^2^ Activation of TLR4 by ligands of sterile inflammation has been demonstrated in various *in vitro/in vivo* models.^2,5,6^ As oxidised forms of lipoproteins might play a lead role in development of CVD, in the next series of experiments we tested the potential of FP7 to modulate TLR4 signalling in response to hydroperoxide-rich oxidised LDL (oxLDL) in THP-1-derived macrophages. It has been shown that oxidised forms of LDL can affect different TLRs, therefore, we compared these effects with LPS, a specific TLR4 activator. Initially, tetrazolium dye (MTT) results revealed that FP7 (10 μM) and oxLDL (up to 100 μg/ml protein) did not have an impact on cell viability (Supplemental Figure 1). In the next series of experiments our results documented the ability of oxLDL, but not native LDL, to induce production of IL-8 and IL-1β in a concentration-dependent manner in THP-1-derived macrophages (Figure 2c and d). In contrast, pre-treatment with FP7 (10 μM) efficiently inhibited production of oxLDL-driven IL-8 and IL-1β production in THP-1-derived macrophages. These results clearly demonstrated the potential of FP7 to negatively regulate TLR4 signalling in response to ligands of sterile and non-sterile inflammation in human THP-1-derived macrophages.

### FP7 inhibits LPS-induced TLR4 signalling in mouse RAW-264.7 macrophages

**Figure 3.**
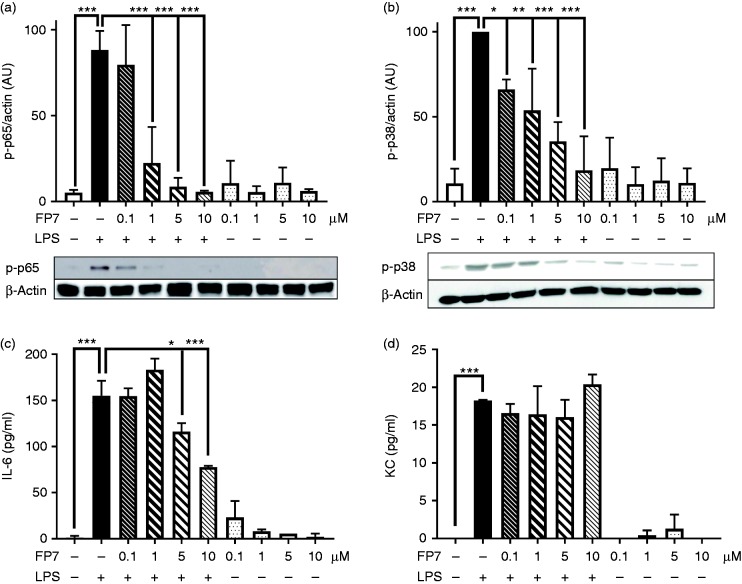
FP7 inhibits LPS-induced TLR4 signalling in mouse RAW-264.7 macrophages. Cells were pre-incubated with FP7 (0–10 μM) for 1 h and then exposed to LPS (100 ng/ml) for 60 min ((a) and (b)) or 16 h ((c) and (d)). Cell lysates were analysed for p65 NF-kB (a), p38 MAPK (b) phosphorylation, and cell media were analysed for IL-6 (c) and keratinocyte chemoattractant (KC) expression (d) using immunoblotting and ELISA analyses, respectively. Actin was used as a loading control. Data are mean ± SD, *n* = 3 at each data point. Significant results are indicated as *P < 0.05, **P < 0.01 and ***P < 0.001.

There is a good body of evidence that protein sequences of human TLR4 and MD-2 are not completely conserved across species and that might reflect the functional activity of the receptor in response to distinct ligands including immune modulators.^19^ This can present real difficulties in validating immune-modulators in preclinical models which can affect human but not mouse TLR4. Having shown that FP7 can inhibit human TLR4, in the next series of experiments we tested the ability of FP7 to affect mouse TLR4 functional activity in mouse RAW-264.7 macrophages. We applied the same experimental design as with THP-1 cells, based on two readouts (activation of TLR4 second messengers and production of TLR4-dependent pro-inflammatory proteins). Initially, FP7 (up to 10 μM) did not affect cell viability (Supplemental Figure 1). Immunoblotting data revealed that FP7 significantly inhibited LPS/TLR4-induced p65 NF-kB and p38 MAPK phosphorylation in a concentration-dependent manner (Figure 3a and b), which was associated with down-regulation of TLR4-dependent cytokine IL-6 (Figure 3c). Interestingly, we found that FP7 was not able to affect the production of LPS-driven KC (mouse IL-8) in mouse macrophages, suggesting that FP7 displayed a selective inhibitory effect between human and mouse TLR4. These results clearly validated the potential of this small molecule to negatively regulate mouse TLR4 signalling, suggesting its application in rodent models for treatment of inflammatory-based CVD.

### FP7 negatively regulates LPS-induced TLR4 signalling in HUVEC

**Figure 4.**
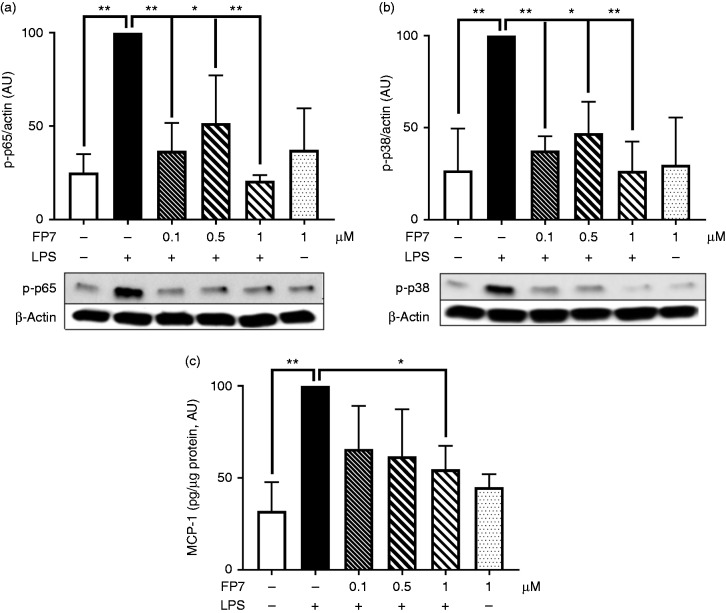
FP7 inhibits LPS-induced TLR4 signalling in HUVEC. Cells were pre-incubated with FP7 (0–5 μM) for 1 h and then exposed to LPS (100 ng/ml) for 60 min ((a) and (b)) or 16 h (c). Cell lysates were analysed for p65 NF-kB (a) and p38 MAPK (b) phosphorylation and monocyte chemoattractant protein (MCP)-1 (c) expression using immunoblotting and ELISA analyses, respectively. Actin was used as a loading control. Data are mean ± SD, *n* = 3 at each data point, Significant results are indicated as *P < 0.05 and **P < 0.01.

Vascular endothelial TLR4 has been shown to play a critical role in initiation and progression of CVD. Endothelial cells use TLR4 signalling to produce pro-inflammatory proteins which initiate an inflammatory process by activating and attracting haematopoietic cells such as monocytes, macrophages or neutrophils. To test the potential of FP7 to modulate endothelial TLR4 signalling pathways, we utilised HUVEC as an *in vitro* model. To determine the effect of FP7 on LPS-induced TLR4 signalling in HUVEC, we analysed the activation of p38 MAPK/p65 NF-kB as second messengers in TLR4 signalling and production of MCP-1, a well-known TLR4-dependent chemokine produced from endothelial cells in response to LPS. Initially, Western blot data revealed that p38 MAPK and p65 NF-kB phosphorylation were elevated in response to LPS in HUVEC (Figure 4a and b). In contrast, FP7 significantly inhibited LPS-stimulated p38 MAPK/p65 NF-kB phosphorylation. Further, ELISA results demonstrated that FP7 blocked LPS-driven MCP-1 expression in HUVEC (Figure 4c). Overall, these data demonstrated that FP7 was a negative regulator of TLR4 signalling in HUVEC.

### FP7 down-regulates TLR4 signalling in mouse aorta in response to sterile inflammation

**Figure 5.**
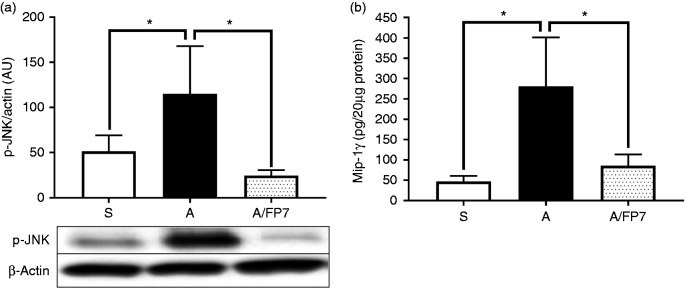
FP7 inhibits Angiotensin II-induced c-Jun N-terminal kinase (JNK) phosphorylation and macrophage inflammatory protein (MIP)-1γ expression in the mouse aorta. Apolipoprotein E-deficient mice were divided into three groups: a sham control negative group (S), an Angiotensin II group (A), and an Angiotensin II/FP7 co-treated group (A/FP7). FP7 (3 mg/kg/d in 50 μl Lipodisq™) was administered s.c. up to 72 h. Tissue samples from mouse aorta were prepared at 72 h, and soluble proteins were analysed for JNK phosphorylation (a) and MIP-1γ expression (b) using immunoblotting and ELISA analyses, respectively. Actin was used as a loading control. Data are mean ± SD, *n* = 3/4 mice at each data point, *P < 0.05.

Prevention of experimental atherosclerosis, aneurysm and heart failure by deletion of TLR4 was previously reported, suggesting that TLR4 may represent a novel therapeutic target for pharmacological treatment of CVD.^8^ Having shown that FP7 can negatively regulate mouse TLR4 signalling, in the next series of experiments we tested the efficacy of FP7 to modulate *in vivo* vascular TLR4 signalling pathways. For this purpose, we utilised the Angiotensin II-infusion in the Apo E-deficient mouse as an *in vivo* model. We have previously reported that the infusion of Angiotensin II in Apo E-deficient mice induced an inflammatory process which was associated with activation of TLR4 signalling and production of pro-inflammatory proteins which peaked at 72 h in mouse aorta.^17,20^ Therefore, we investigated the effect of FP7 on TLR4 signalling in the mouse aorta at 72 h of Angiotensin II infusion. Bearing in mind the poor solubility of FP7 in aqueous solutions, we used a Lipodisq™ carrier technology for an *in vivo* administration of FP7 in Apo E-deficient mice. The results from Western blot analysis of proteins isolated from the mouse aorta revealed the ability of FP7 to significantly inhibit Angiotensin II-stimulated JNK phosphorylation, a well-known mediator of TLR4 signalling (Figure 5a). Further, we explored whether FP7 could have an impact on Angiotensin II-driven production of pro-inflammatory proteins in the mouse aorta. Mouse tissue lysates were analysed on a mouse inflammation Ab array (containing 40 pro-inflammatory proteins). The semi-quantitative analysis demonstrated that FP7 inhibited, to various extents, the expression of 15/25 Angiotensin II-driven pro-inflammatory proteins (Supplemental Table 1). Finally, we validated the blocking effect of FP7 on MIP-1γ production (a well-known chemokine for attraction and differentiation of circulating monocytes). ELISA results showed that FP7 greatly decreased production of MIP-1γ in the mouse aorta (Figure 5b). These data demonstrated that FP7 negatively regulated mouse TLR4 signalling (*in vivo)*, suggesting that this molecule could be successfully used in preclinical models for treatment of CVD.

## Discussion

Inflammation has been documented as a critical event in a variety of CVD. In this regard, a number of studies have shown the essential role of TLR4 in several cardiovascular pathologies, suggesting that the modulation of TLR4 signalling pathways will be beneficial for treatment.^21,22^ Pharmacological intervention using TLR4 antagonists has been a challenging approach for the last two decades; however, these candidates failed in different stages of clinical trials and therefore a generation of new TLR4 modulators is of great interest.^11,12^

Recently, it has been reported that the MD-2-directed synthetic TLR4 antagonist FP7 inhibited TLR4 function and glycolytic re-programming of dendritic cells, and protected mice from death due to TLR4-dependent influenza infection.^23^ In this study we showed that FP7 had the potential to inhibit haematopoietic and non-haematopoietic TLR4 signalling in response to distinct TLR4 ligands which are associated with the pathogenesis of CVD.

TLR4 plays an important role in prolonged and sustained activation of the monocytes/macrophage system which is fundamental for initiation and progression of inflammatory diseases. In this study we demonstrated the potential of the TLR4 antagonist FP7 to block MyD88-dependent TLR4 signalling both in human monocytes and macrophage. We showed the ability of FP7 to negatively regulate TLR4 signalling to second messengers (p65 NF-kB/p38 MAPK) and pro-inflammatory proteins secretion (IL-8, IL-6, IL-1β, MIP-1α). Importantly, we also showed that irrespective of the time of administration (prior, simultaneously or after LPS stimulation), this small molecule inhibited the production of LPS/TLR4-driven pro-inflammatory cytokines (IL-6 and TNF-α). These *in vitro* results complemented the data obtained from an *in vivo* experimental model, where co-administration of FP7 and Angiotensin II infusion blocked TLR4 functional activity in Apo E-deficient mouse aortas. While the *in vitro* effect of pre-incubation or co-incubation with FP7 could be explained by competition between LPS and FP7 to bind MD-2 and displaced LPS,^24^ the blocking effect of FP7 administered after LPS requires a different explanation as the LPS/MD-2/TLR4 active complex can activate the second messengers (MAPK or NF-kB) in a few minutes. Further studies to investigate the effect of FP7 on downstream targets in TLR4 signalling, including TLR4 internalisation and degradation, are needed.

We report that this small molecule could negatively modulate TLR4 activation in response to ligands of non-sterile (LPS) and sterile inflammation (oxLDL), showing that FP7 blocks TLR4-mediated inflammatory processes triggered by distinct TLR4-associated danger signals.

Although the protein sequence of TLR4 is conserved across species, the sequence of the MD-2 adaptor is different, so that species-specific ligand discrimination or adaptor selection has been observed between human and mouse TLR4.^19^ For certain synthetic and natural lipid A variants, such as lipid IVa, when transferring from the human to murine TLR4/MD-2/CD14 system, an antagonistic effect could switch to an agonistic effect.^25^ The species-specificity is due to different positioning of the same ligand, thus causing differential activity.^24^ Our data strongly confirm the ability of FP7 to block both human and mouse TLR4 signalling in macrophages. In this study we demonstrated the efficacy of FP7 to negatively regulate TLR4 signalling in different haematopoietic cells. The fact that FP7 was shown to block TLR4/MD-2/CD14 interactions based on its high affinity to bind MD-2 may explain the specific biochemical properties of this small molecule.^23,24^

We and other groups have found that early stages of vascular disease development are associated with activation of MAPK and production of pro-inflammatory proteins in the experimental model of an Angiotensin II infusion in hypercholesterolaemic mice.^20,26^ TLR4 signalling plays an essential role in propagation of inflammation and mediates production of a large proportion of the pro-inflammatory proteins production. It has been reported that TLR4 deficiency attenuated aneurysm and atherosclerosis development, suggesting that TLR4 signalling is fundamental in related vascular pathologies.^8^ TLR4 signalling exerted effects through non-haematopoietic cell types, suggesting that vascular cells might use the TLR4 signalling network in response to an inflammatory environment.^8^ In this regard we have previously reported that another synthetic TLR4 antagonist (IAXO-102) inhibited TLR4 signalling in HUVEC and protected against experimental abdominal aortic aneurysm development.^20^ In this study we showed the potential of the synthetic TLR4 antagonist FP7 to block TLR4 signalling in non-haematopoietic vascular cells.

Having shown that FP7 can affect mouse TLR4, further we examined the potential of FP7 to modulate *in vivo* TLR4 signalling utilising a well-established model of an Angiotensin II infusion in the hypercholesterolaemic Apo E-deficient mouse. Using the nano-carrier Lipodisq™, we were able to deliver FP7 by improving the compound’s solubility in aqueous and physiological solvents. We have successfully used a similar nano-carrier approach for administration of IAXO-102 (TLR4 antagonist) with a poor solubility, for which pharmacokinetic studies demonstrated that the dose of 3 mg/kg/d was sufficient to produce positive signal and drug distribution among several organs.^20^

Several studies from the literature, including our data, prove the role of MAPK in CVD development.^19,20,24^ JNK has been shown as an important target for Angiotensin II/TLR4 signalling leading to activation of c-jun/c-fos and production of a number of pro-inflammatory proteins. In this regard, it has been reported that inhibition of JNK markedly affects the initiation and progression of CVD.^27,28^ Previously, we have identified JNK as a target by which rosiglitazone and IAXO-102 inhibited Angiotensin II/TLR4-induced inflammatory responses in the mouse aorta and reduced markedly aortic aneurysm formation.^17,20^ In this study FP7 efficiently inhibited JNK phosphorylation and negatively regulated a large number of TLR4-dependent pro-inflammatory proteins in the mouse aorta. These results clearly demonstrated the ability of FP7 to block the initiation of Angiotensin II-driven sterile inflammation in the mouse aorta. Further studies using histological and immunohistochemistry approaches to demonstrate the impact of FP7 on aortic tissue remodelling and identification of specific cells responsible for a propagation of TLR4 signalling at late stages of the inflammatory process in the Angiotensin II-infused Apo E-deficient mouse model are needed.

In support to our findings, Perrin-Cocon and colleagues recently reported that FP7 can block TLR4 activity in response to another trigger of sterile inflammation (high mobility group box 1 protein, HMGB-1) in dendritic cells.^23^ Furthermore, using the dextran-sulphate-sodium-induced rodent model of colitis, we recently demonstrated that FP7 efficiently blocks the inflammatory process in this preclinical model of inflammatory bowel disease (unpublished data). In the literature, it has been shown that Angiotensin II infusion may trigger sterile inflammation by generation of reactive oxygen species leading to formation of oxLDL in hyperlipidaemic mice.^29^ In relation of this, we suggested that FP7 might inhibit TLR4 signalling activated by ligands of ‘sterile’ inflammation, such as oxLDL, in the mouse model infused with Angiotensin II. In support of this notion, our data showed that this small molecule inhibited TLR4 signalling in response to oxLDL in human THP-1-derived macrophages. This is an important issue because different TLR4 ligands have a specific signature in production of TLR4-dependent proteins leading to various outcomes. Overall, these data confirmed the potential of FP7 to negatively modulate *in vitro* and *in vivo* TLR4 signalling in response to DAMPs-triggered sterile inflammation. Furthermore, this study also shows the potential of FP7 to inhibit TLR4 signalling in response to distinct TLR4 ligands such as LPS or oxLDL.

In conclusion, the results from this study demonstrated that the synthetic TLR4 antagonist FP7 was effective in blocking haematopoietic and non-haematopoietic vascular TLR4 signalling, suggesting the potential of this small molecule for pharmacological intervention of CVD and other inflammatory diseases. Future work, based on the experimental model (*in vivo*) used in this study, will be focused on preclinical validation of FP7 for treatment of atherosclerosis and aneurysms.

## Supplemental Material

Supplemental material for The synthetic glycolipid-based TLR4 antagonist FP7 negatively regulates *in vitro* and *in vivo* haematopoietic and non-haematopoietic vascular TLR4 signalling Click here for additional data file. Supplemental material for The synthetic glycolipid-based TLR4 antagonist FP7 negatively regulates *in vitro* and *in vivo* haematopoietic and non-haematopoietic vascular TLR4 signalling by Charys Palmer, Francesco Peri, Frank Neumann, Feroz Ahmad, David S. Leake and Grisha Pirianov in Innate Immunity
