# Supplementary material for: The synthetic glycolipid-based TLR4 antagonist FP7 negatively regulates in vitro and in vivo haematopoietic and non-haematopoietic vascular TLR4 signalling
Source: Innate Immun. 2018 Sep 12;24(7):411–21. doi: 10.1177/1753425918798904 (PMC6830872; doi:10.1177/1753425918798904)
Supplement: Supplemental material for The synthetic glycolipid-based TLR4 antagonist FP7 negatively regulates in vitro and in vivo haematopoietic and non-haematopoietic vascular TLR4 signalling [file Supplemental_material2.pdf]

Supplementary Materials

Table S1. FP7 modulates expression of Angiotensin II-driven proteins in mouse aorta. Apo E deficient mice were divided into three groups: sham control negative group, Angiotensin II group, Angiotensin II/FP7 co-treated group. FP7 (3 mg/kg/d) in Lipodisq^{TM}) was administered s.c. up to 72 h. Tissue samples from supra-renal aorta were prepared at 72 h, and soluble proteins were semi-quantitatively analysed on a mouse Inflammation Ab array (Ray Bio Tech) following manufacturer's instructions. Data are normalized to array controls and expressed as a fold change in comparison to untreated controls. FP7 down-regulates TLR4-dependent pro-inflammatory proteins (* at least 50% reduction in expression of pro-inflammatory proteins compared to Angiotensin II).

Pro-inflammatory proteins	Sham	AngII	AngII FP7	Pro-inflammatory proteins	Sham	AngII	AngII FP7
1. BLC	1.0	0.92	1.01	21. IL-17	1.0	2.86	1.26*
2. CD80L	1.0	1.90	1.63	22. I-TAC	1.0	2.28	1.61
3. Eotaxin 1	1.0	3.01	2.31	23. KC	1.0	2.40	1.11*
4. Eotaxin 2	1.0	1.53	1.38	24. Leptin	1.0	2.56	1.70*
5. FasL	1.0	2.28	3.09	25. LIX	1.0	1.02	0.76
6. Fractalkine	1.0	2.55	2.40	26.Lymphotactin	1.0	0.85	0.70
7. GCSF	1.0	1.29	1.12	27. MCP-1	1.0	1.07	0.85
8. GMCSF	1.0	2.62	1.08*	28. MCSF	1.0	0.98	0.75
i9. INF-γ	1.0	3.84	2.18*	29. MG	1.0	1.35	1.42
10. IL-1α	1.0	2.61	1.53*	30. MIP-1α	1.0	1.02	0.92
11. IL-1β	1.0	2.31	1.37*	31. MIP-1γ	1.0	3.37	0.88*
12. IL-2	1.0	0.89	0.75	32. RANTES	1.0	5.03	0.95*

13. IL-3	1.0	1.43	1.43	33. SDF-1	1.0	2.32	1.01*
14. IL-4	1.0	1.32	0.81	34. TCA-3	1.0	1.55	0.70*
15. IL-6	1.0	1.35	1.36	35. TECK	1.0	2.50	1.40*
16. IL-9	1.0	1.56	1.16	36. TIMP-1	1.0	1.97	0.94*
17. IL-10	1.0	1.78	1.65	37. TIMP-2	1.0	2.00	0.1.38
18. IL-12p40	1.0	2.69	2.26	38. TNF-α	1.0	1.90	1.15*
19. IL-12p70	1.0	1.14	0.95	39. TNFRI	1.0	1.20	0.80
20. IL-13	1.0	3.17	1.09*	40. TNFRII	1.0	1.02	0.55

BLC - B lymphocyte chemoattractant (CXCL13)

CD80L- Cluster of Differentiation 80

Eotaxin-1 – Eosinophils chemokine (CCL11)

Eotaxin-2 - Eosinophils chemokine (CCL24)

FasL – Cluster of differentiation 95 ligand (CD95L)

Fractalkine – (CX3CL1)

GCSF - Granulocyte colony-stimulating factor

GMCSF - Granulocyte-macrophage colony-stimulating factor

I-TAC - Interferon-inducible T-cell chemoattractant (CXCL11)

KC - Keratinocyte-derived chemokine

Leptin - Adipose derived hormone

LIX - Epithelial-derived neutrophil-activating peptide 78 (CXCL5)

Limphotactin – LTN (XCL1)

MCP-1 - Monocyte chemotactic protein-1 (CCL2)

MIP-1α - Macrophage inflammatory protein-1 (CCL3)

MIP-1γ - Macrophage inflammatory protein 1 gamma (CCL9/10)

RANTES - Regulated upon Activation, Normal T-cell Expressed, and Secreted (CCL5)

SDF-1 - Stromal cell-derived factor-1 (CXCL12)

TCA-3 – T cell activation gene-3 (CCL1)

TECK - Thymus-Expressed Chemokine (CCL25)

TIMP-1 - Metallopeptidase inhibitor 1;

TIMP-2 - Metallopeptidase inhibitor 2

TNFRI - TNF receptor-1

TNFRII- TNF receptor-II

Supplementary figures

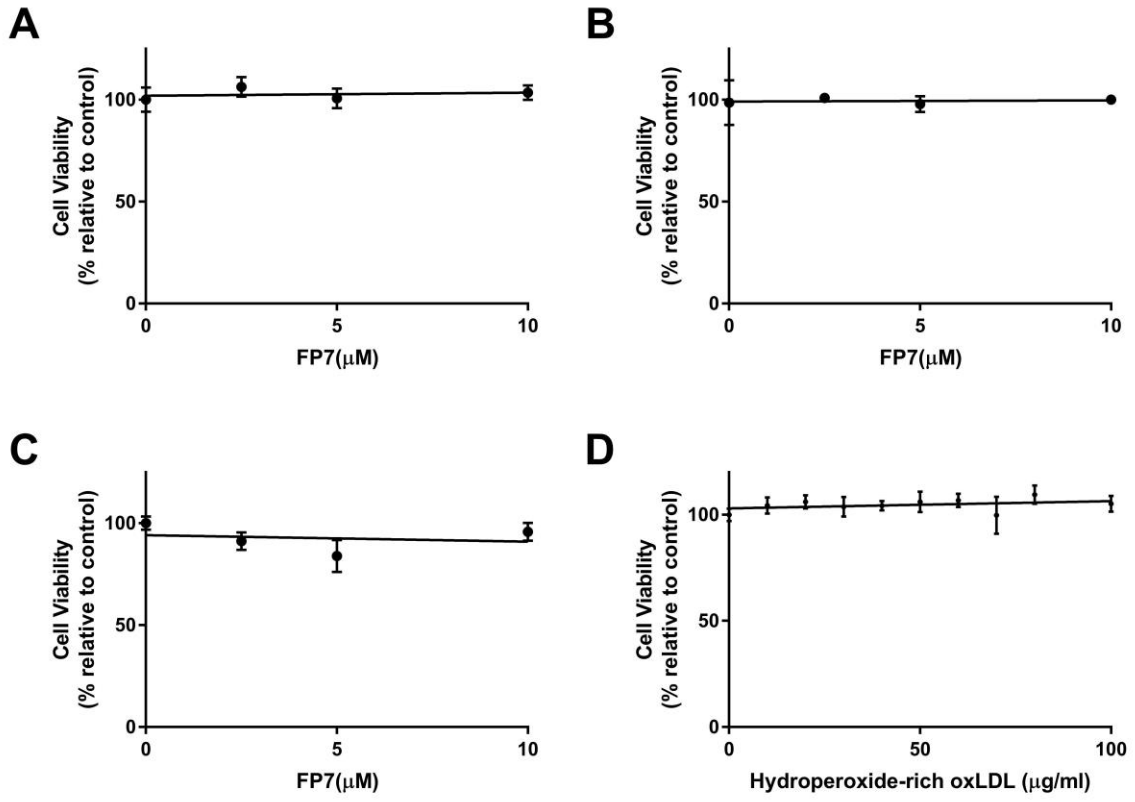

Figure S1. Effect of FP7 on cell viability. THP-1 cells (A), THP-1-derived macrophages (B and D) and RAW-264.7 cells (C) were treated with FP7 (0-10 μM) or hydoxLDL (0-100 μg/ml protein) for 24 h. Cell viability was measured by a MTT assay. Data are mean ± SD, *n* = 6 at each data point. No statistically significant results were obtained.

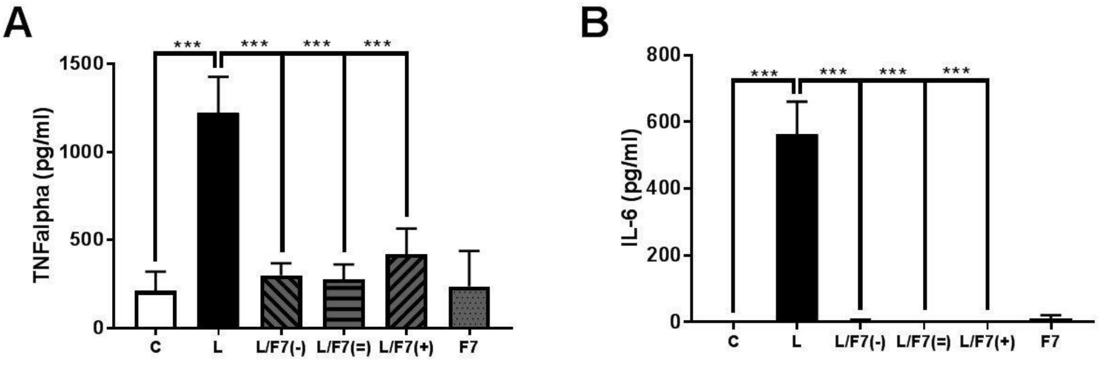

Figure S2. Irrespective of the time of administration FP7 reduces LPS-induced IL-6 and TNF-α production in THP-1 macrophages. THP-1 macrophages were treated with FP7 (F7) (10 μM) 0.5 h prior (-), simultaneously (=) or 0.5 h post (+) LPS (L) (10 ng/ml) exposure. Culture medium was collected after 18 h and analysed via ELISA for TNF-α (A) and IL-6 (B) production. Data are mean ± SD of n = 3 experiments. Significant results are shown as ****P* < 0.001.
